# New Insights into Chemical and Biological Properties of Funicone-like Compounds

**DOI:** 10.3390/toxins14070466

**Published:** 2022-07-08

**Authors:** Maria Michela Salvatore, Marina DellaGreca, Anna Andolfi, Rosario Nicoletti

**Affiliations:** 1 Department of Chemical Sciences, University of Naples Federico II, 80126 Naples, Italy; mariamichela.salvatore@unina.it (M.M.S.); dellagre@unina.it (M.DG.); 2 Institute for Sustainable Plant Protection, National Research Council, 80055 Portici, Italy; 3 BAT Center—Interuniversity Center for Studies on Bioinspired Agro-Environmental Technology, University of Naples Federico II, 80055 Portici, Italy; 4 Department of Agricultural Sciences, University of Naples Federico II, 80055 Portici, Italy; rosario.nicoletti@crea.gov.it; 5 Council for Agricultural Research and Economics, Research Center for Olive, Fruit, and Citrus Crops, 81100 Caserta, Italy

**Keywords:** fungal metabolites, natural products, *Talaromyces*, *Penicillium*, secondary metabolites, mycotoxins

## Abstract

Funicone-like compounds are a homogeneous group of polyketides that, so far, have only been reported as fungal secondary metabolites. In particular, species in the genus *Talaromyces* seem to be the most typical producers of this group of secondary metabolites. The molecular structure of funicone, the archetype of these products, is characterized by a γ-pyrone ring linked through a ketone group to a α-resorcylic acid nucleus. This review provides an update on the current knowledge on the chemistry of funicone-like compounds, with special emphasis on their classification, occurrence, and diverse biological activities. In addition, their potential relevance as mycotoxins is discussed.

## 1. Introduction

Research on fungal secondary metabolites is mainly driven by remarks concerning their bioactive properties, which can either be inherent to their role in biocenotic interrelations or their effects on human health, the latter depending on either their possible accumulation in foodstuffs as mycotoxins, or eventual pharmaceutical relevance.

Funicones and structurally related compounds represent a homogeneous group of fungal polyketides that were initially characterized as determinants of the antagonistic abilities by the producers against other microorganisms, but were later found to possess remarkable biological properties that have promoted their consideration as drug prospects. Considering that these properties are partly based on observations concerning cytostatic and antiproliferative effects on human cells, these products should be also evaluated with reference to toxicological aspects related to possible contamination of foodstuffs by the producing fungi.

In light of the novel knowledge developed in over a decade since the publication of a previous review [1], this paper offers an update on the state of the art concerning occurrence, bioactivities, structural, synthetic, and biosynthetic aspects of funicone-like compounds.

## 2. Structures and Chemical Properties

Funicone-like compounds include natural products characterized by a molecular structure that is built on a γ-pyrone ring linked through a ketone group to a α-resorcylic acid nucleus. A total of 34 chemically defined compounds, which are referable to this basic structural model, have been identified and characterized so far. Among them, 13 can be considered true funicones because the typical moieties are present without alterations. The other compounds, showing modifications on the α-resorcylic acid nucleus, on the γ-pyrone ring, or on both moieties, are grouped in three subclasses, namely phthalide, furopyrone, and pyridone types, depending on peculiar substructural variations (Table 1).

### 2.1. True Funicones

**Figure 1:**
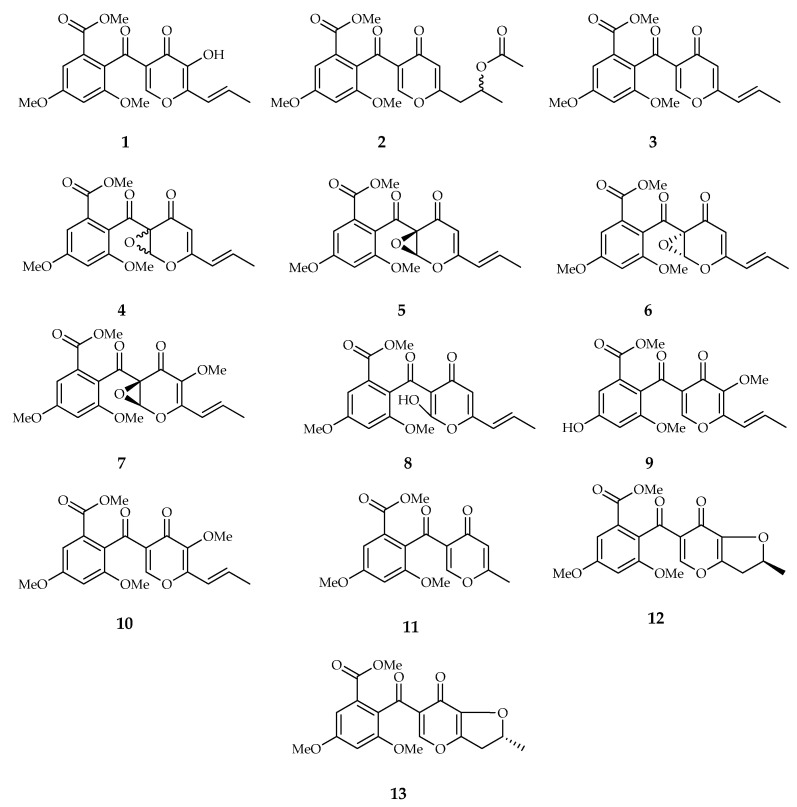
Structures of true funicones (**1**–**13**): funicone, actofunicone, deoxyfunicone, 9,14-epoxy-11-deoxyfunicone, 9*R*,14*S*-epoxy-11-deoxyfunicone, 9*S*,14*R*-epoxy-11-deoxyfunicone, 3-*O*-methyl-5,6-epoxyfunicone, 6-hydroxyl-deoxyfunicone, isofunicone, 3-*O*-methylfunicone, rapicone, pinophilone A, and pinophilone B.

In temporal terms, funicone [benzoic acid, 2-[[5-hydroxy-4-oxo-6-(1E)-1-propenyl-4H-pyran-3-yl]carbonyl]-3,5-dimethoxy, methyl ester] (**1**) is the founder of this group of compounds, originally characterized from a culture of *Penicillium funiculosum* [2]. Subsequently, a structural isomer, namely isofunicone (**9**) [16], and several derivatives, which differ from the parent compound by few substitutions, were identified (Figure 1). This subclass also includes some epoxide derivatives (**4**–**7**) on the γ-pyrone ring, two of them (**5**,**6**) isolated from co-cultures of a strain of *Penicillium* sp. with the actinomycete *Streptomyces fradiae* [14]. Pinophilones A and B (**12** and **13**) are the only funicone-like compounds presenting a dihydrofuran fragment obtained from the cyclization of the hydroxyl group on the γ-pyrone ring and the double bond on the propenyl chain [28].

**Figure 2:**
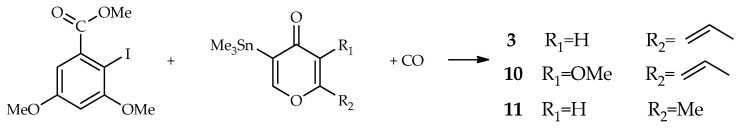
General procedures for synthesis of funicones.

The rising interest of the scientific community in these substances has led to the development of approaches for their synthesis. In particular, deoxyfunicone (**3**), 3-*O*-methylfunicone (**10**) [52], and rapicone (**11**) [53] were efficiently prepared by carbonylative Stille cross-coupling reactions between methyl 2-iodo-3,5-dimethoxybenzoate and functionalized γ-pyrone (Figure 2). 5-Stannane derivatives were prepared starting from commercially available kojic acid in four steps [52,53].

### 2.2. Furopyrone Type

**Figure 3:**
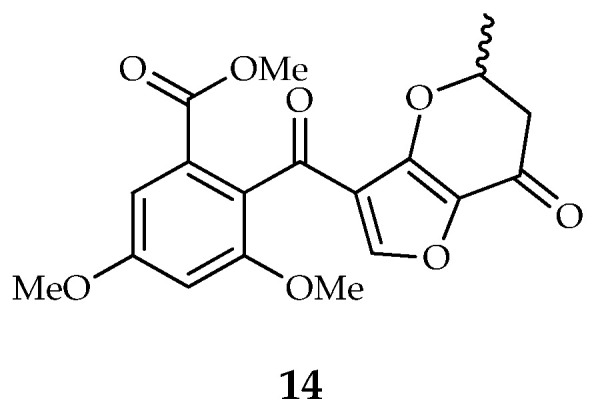
Structure of penifupyrone (**14**).

Penifupyrone (**14**) is the only member of the furopyrone type carrying a 5H-furo[3,2-b]pyran-7(6H)-one moiety instead of a γ-pyrone ring (Figure 3). It was isolated for the first time from an endophytic strain of *Talaromyces* sp., along with funicone, deoxyfunicone, and 3-*O*-methylfunicone [5].

### 2.3. Phthalide Type

**Figure 4:**
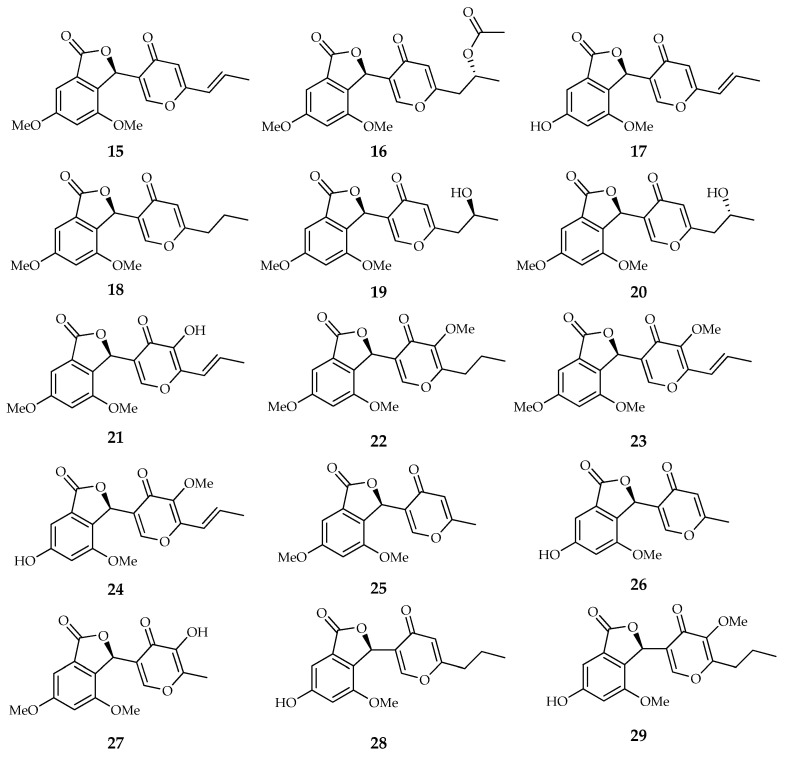
Structures of compounds from the phthalide type (**15**–**29**): vermistatin, acetoxydihydrovermistatin, 6-demethylvermistatin, 14,15-dihydrovermistatin, 2″-epihydroxydihydrovermistatin, hydroxydihydrovermistatin, hydroxyvermistatin, 5′-*O*-methyldihydrovermistatin, methoxyvermistatin, neosarphenol A, penisimplicissin, 6-demethylpenisimplicissin, 5′-hydroxypenisimplicissin, pinophilone C, and pinophilone D.

The molecular structure of compounds in this subclass includes a 4,6-dimethoxyphthalide moiety (Figure 4). Vermistatin (**15**) is the reference compound of this group, deriving its name from a strain of *Talaromyces flavus* identified in anamorphic-stage *Penicillium vermiculatum* [47]. This metabolite was later isolated as a product of *Pseudocercospora* (=*Mycosphaerella*) *fijiensis* and wrongly reported as a new compound with the name fijiensin [30]. This is not surprising because the attribution of different names to the same chemical structure represents a recurring nomenclatural issue in natural product research [54].

Based on the currently available data, vermistatin represents the most frequent funicone-like compound, having been extracted as a product of at least 15 species. It is frequently extracted along with some derivatives, such as hydroxy- (**21**) and methoxyvermistatin (**22**), 6-demethylvermistatin (**17**), 14,15-dihydrovermistatin (**18**), hydroxy- (**20**) and acetoxy-dihydrovermistatin (**16**), and penisimplicissin (**25**) [6,7,21,28,33,34,45,49].

Neosarphenol (**24**) is an isomer of hydroxyvermistatin, which was named on the basis of the producing fungus, *Neosartorya glabra* (currently reclassified as *Aspergillus neoglaber*), rather than with reference to its chemical structure [40].

### 2.4. Pyridone Type

**Figure 5:**
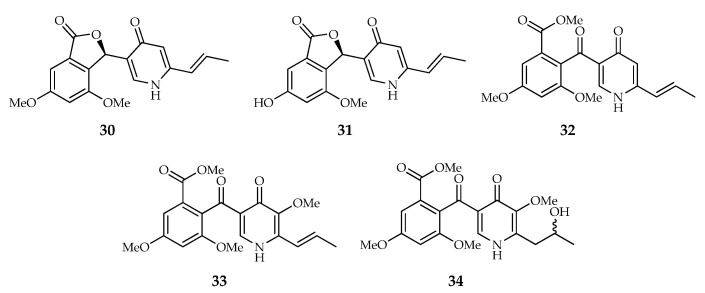
Structures of compounds from the pyridone type (**30**–**34**): penicidone A–D and talarodone A.

This series includes compounds containing a γ-pyridone moiety. The molecular structures of penicidone A and B (**30**,**31**) are characterized by the presence of an α-resorcylic acid moiety linked through a ketone group to a γ-pyridone, whereas penicidone C, D and talarodone A (**32**–**34**) contain the typical 4,6-dimethoxyphthalide moiety of vermistatin replacing the α-resorcylic acid nucleus (Figure 5). Nevertheless, Murakami et al. [18] represented penicidone D (**33**) in γ-pyridol form, instead of γ-pyridone form.

## 3. Fungal Sources

The data summarized in Table 2 show that the fungi reported as funicone producers have been recovered from various substrates, often in association with plants or other organisms, and in diverse environments, both terrestrial and marine. They are also quite heterogeneous in taxonomic terms, as they belong to two Ascomycetes classes: the Dothideomycetes and Eurotiomycetes. Members in the first class are sparse, being ascribed to five orders, with each of them represented by a single strain. Even considering the approximate taxonomic identification of three strains, which were only identified at the genus level, it is clear that funicone biosynthetic aptitudes occur among Dothideomycetes, and might be more widespread than currently known. Conversely, the Eurotiomycetes look to be much more abiding producers and taxonomically homogeneous, with about 31 strains belonging to three genera in two families. Again, some uncertainty in identification is to be noted, deriving from the absence of adequate support by sequencing of valid DNA markers, and by the provisional ascription to *Penicillium* sp. of some strains prior to the formal separation of the biverticillate *Penicillium* species and their assignment to the genus *Talaromyces* [55]. In this respect, the identification of strain IFM53375 as *Penicillium simplicissimum* was considered unreliable by leading taxonomists of these fungi based on a secondary metabolite profile more respondent to *Talaromyces* [55]. In another case, the producing strain (AF1-2) was not identified at all [26]; however, the image provided by the authors showing its bright yellow mycelium and the overlying green sporulation in culture on agar medium unequivocally allows its ascription to *Talaromyces*. In any case, species in the genus *Talaromyces* are the most typical producers of funicone-like compounds; with reference to the recent affirmation of the horizontal gene transfer concept [56,57], it cannot be excluded that the other fungal species may have occasionally acquired their funicone-biosynthetic abilities through this intriguing biological mechanism.

Recently, some independent studies have reported that production of funicone-like compounds may occur in co-cultures of various microbial strains (Table 3). Again, the Eurotiomycetes are more represented in these few studies, and can be thought to provide the genetic base for biosynthesis, which is eventually stimulated by the co-cultured strain in the course of an antibiotic struggle, as clearly demonstrated in the case of the pairing between *Talaromyces siamensis* and *Phomopsis* sp. (Sordariomycetes, Diaporthaceae) [43]. In two cases, the partner microbe was represented by *Streptomyces* strains (Actinomycetota), which are well-known for their capacity to modulate the metabolic potential of fungi [60].

## 4. Biosynthesis

**Figure 6:**
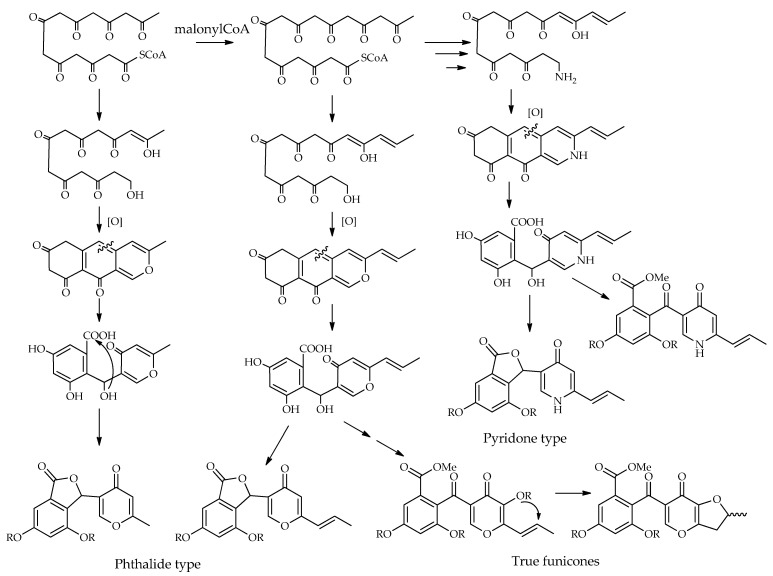
Proposed biosynthetic schemes of funicone-like compounds.

The potential biosynthetic pathways of funicone-like compounds have been investigated by two independent research groups [21,28]. Figure 6 shows a possible scheme for each type of compound proposed in the previous section. Funicone-like compounds are epta and octaketides, originating from units of acetate-mevalonate. The main structural differences can be caused by the folding of the eptaketidic and octaketidic chains, which produce structures with a methyl or a propenyl group, respectively, on the γ-pyrone ring. The presence of an amino group in compounds belonging to the pyridone type suggests a possible transamination process during the biosynthesis of γ-pyridone. The origin of the phthalide type can be attributed to the lactonization of the carboxylic group in the α-resorcylic ring, with the hydroxyl group produced through the reduction of the exocyclic ketone group.

Subsequent functional modifications (e.g., reduction, epoxydation, hydroxylation, methylation, and acetylation) are responsible for the ample structural variability observed in the group of funicone-like compounds.

## 5. Bioactivities

As previously introduced, the biological activity of funicones was initially evaluated with reference to antibiotic properties, generally evidencing poor effects against bacteria and yeasts, and more relevant activities against filamentous fungi. Subsequent investigations on antiproliferative properties against human cells line have become prevalent, underlining the potential of these compounds as antitumor drugs. Additional data have been gathered on the antiviral and the insecticidal properties, and the inhibitory effects toward several enzymes; moreover, some minor bioactivities have been described. The outcomes of this wide-ranging investigational work, as assessed in quantitative terms, are summarized in Table 4.

## 6. Potential Role of Funicone-like Compounds as Mycotoxins

The applicative aspects of studies concerning fungal bioactive secondary metabolites involve their accumulation in food products and ensuing possible impact on consumers’ health. Within the multitude of such compounds described so far, a very small number have been considered mycotoxins, based on the results of toxicological studies that noted their noxious effects on humans and animals [69]. This implies that a high number of compounds yet to be examined for these aspects may represent a potentially underestimated concern [70,71].

Funicones are one of the classes of fungal secondary metabolites for which very limited assessments have been carried out in this regard so far. Most of the producing species are not established pathogens of crops, with the exception of *Pseudocercospora* (=*Mycosphaerella*) *fijiensis*, a vermistatin producer that is known as the agent of black sigatoka disease of banana [72]. However, this is a leaf pathogen that is not known to spread to fruit, implying that it is unlikely that bananas can be contaminated with vermistatin. Nevertheless, a search for this compound in some fruit products carried out in Nigeria evidenced its presence at low levels (0.30 µg kg^−1^) in pineapple and mixed juices [73]. This is not at all surprising, as several *Talaromyces* spp. are commonly found in association with both healthy and diseased pineapples, including *T. purpureogenus*, *T. funiculosus*, and *T. flavus*, which may even survive pasteurization [74,75,76,77]. Conversely, a preliminary search carried out in Italy on marketed pineapple juices yielded negative results with reference to the eventual presence of 3-*O*-methylfunicone [78]. Recently, vermistatin was also detected in the analysis of grains used as cattle and poultry feed in Kenya [79], indicating that it may also occur as a cereal contaminant. Moreover, the finding of vermistatin as a product in co-cultures of strains of *Alternaria alternata* and *Streptomyces exfoliatus* [37] deserves to be further investigated, particularly in view of verifying the biosynthetic capacities by the first species. It is known as a pathogen of many crops and a saprophyte able to proliferate in several kinds of foodstuffs, with very important implications as a mycotoxin producer [80].

Considering the widespread endophytic occurrence of *Talaromyces* spp. [23,81], which are the dominant producers of funicones, the possible release of these compounds in plant products may arise during the postharvest phase, where the biosynthetic aptitudes can be boosted along with the saprophytic development. Recent reports of these fungi as postharvest pathogens concern *T. albobiverticillius* on pomegranate [82], *T. rugulosus* on grapes [83], *T. minioluteus* on onion bulbs and quince, orange, and tomato fruit [84], and both of the latter two species on pears [85]. Although none of these species are known to produce funicones, it is quite possible that other *Talaromyces* spp. producers of these compounds may affect fruit and other crop products, likewise documented for pineapple. This conclusion is supported by the finding of *T. funiculosus* as an agent of fruit core rot of peach [86].

Among the other funicone sources, *Ramichloridium apiculatum*, generally recorded as a soil saprophyte and only known as a producer of rapicone [27], was reported as an agent of sooty blotch and flyspeck of apples and pears in China [87], which may represent an indication for possible contamination of these fruits and their derived transformation products.

## 7. Conclusions

The present review provides an update on the recent developments concerning the distribution, chemical diversity, bioactivity and implications of occurrence of funicone-like compounds. The structures and properties of 34 funicone-like compounds extracted from different fungal species were reviewed. In particular, species in the genus *Talaromyces* seem to be the most typical producers of this group of secondary metabolites, soliciting consideration in view of possible chemotaxonomic implications.

In addition to outlining the general anti-inflammatory, antifungal, antiviral, and cytotoxic activities of these compounds, the available data indicate vermistatin as the most credited candidate to be added to the list of mycotoxins currently considered as food contaminants, with reference to its more common occurrence amongst the known funicone producers. The majority of these taxonomically heterogeneous fungi can perform its biosynthesis, implying that its presence in crop products may be more than just occasional. Whether this represents a threat or, conversely, can eventually be beneficial to consumers’ health based on the described bioactivities, deserves thorough further assessments.

## Figures and Tables

**Table 1:** List of funicone-like compounds gathered from the literature.

Code	Name	Formula	Nominal Mass (U)	Source
**True Funicones**				
**1**	Funicone	C_19_H_18_O_8_	374	[2,3,4,5,6,7,8]
**2**	Actofunicone	C_21_H_22_O_9_	418	[9]
**3**	Deoxyfunicone	C_19_H_18_O_7_	358	[5,7,9,10,11,12,13,14]
**4**	9,14-Epoxy-11-deoxyfunicone	C_19_H_18_O_8_	374	[4]
**5**	9*R*,14*S*-Epoxy-11-deoxyfunicone	C_19_H_18_O_8_	374	[14]
**6**	9*S*,14*R*-Epoxy-11-deoxyfunicone	C_19_H_18_O_8_	374	[14]
**7**	3-*O*-Methyl-5,6-epoxyfunicone	C_20_H_20_O_9_	404	[15]
**8**	6-Hydroxyl-deoxyfunicone	C_19_H_18_O_8_	374	[8]
**9**	Isofunicone	C_19_H_18_O_8_	374	[16]
**10**	3-*O*-Methylfunicone	C_20_H_20_O_8_	388	[5,7,17,18,19,20,21,22,23,24,25,26]
**11**	Rapicone	C_17_H_16_O_7_	332	[27]
**12**	Pinophilone A	C_19_H_18_O_8_	374	[28]
**13**	Pinophilone B	C_19_H_18_O_8_	374	[28]
**Furopyrone type**				
**14**	Penifupyrone	C_19_H_18_O_8_	374	[5,17,18]
**Phthalide type**				
**15**	Vermistatin (=fijiensin)	C_18_H_16_O_6_	328	[3,4,6,7,9,12,20,21,28,29,30,31,32,33,34,35,36,37,38,39,40,41,42,43,44,45,46,47,48]
**16**	Acetoxydihydrovermistatin	C_20_H_20_O_8_	388	[6,33]
**17**	6-Demethylvermistatin	C_17_H_14_O_6_	314	[8,21,28,40,49]
**18**	14,15-Dihydrovermistatin	C_18_H_18_O_6_	330	[6,8,12,28,33,36,38,41,44,45,46]
**19**	2″-epihydroxydihydrovermistatin	C_18_H_18_O_7_	346	[21,28]
**20**	Hydroxydihydrovermistatin	C_18_H_18_O_7_	346	[6,33]
**21**	Hydroxyvermistatin	C_18_H_16_O_7_	344	[7,21,28,34]
**22**	5′-*O*-methyldihydrovermistatin	C_19_H_20_O_7_	360	[28]
**23**	Methoxyvermistatin	C_19_H_18_O_7_	358	[6,7,21,28,34,40,42,50]
**24**	Neosarphenol A	C_18_H_16_O_6_	344	[40]
**25**	Penisimplicissin	C_16_H_14_O_6_	302	[3,6,20,21,28,33,44,45]
**26**	6-Demethylpenisimplicissin	C_15_H_12_O_6_	288	[21,28]
**27**	5′-Hydroxypenisimplicissin	C_16_H_14_O_7_	318	[21]
**28**	Pinophilone C	C_17_H_16_O_6_	316	[28]
**29**	Pinophilone D	C_18_H_18_O_7_	346	[28]
**Pyridone type**				
**30**	Penicidone A	C_18_H_17_NO_5_	327	[51]
**31**	Penicidone B	C_17_H_15_NO_5_	313	[51]
**32**	Penicidone C	C_19_H_19_NO_6_	357	[18,21,28,51]
**33**	Penicidone D	C_20_H_21_NO_7_	387	[17,18,28]
**34**	Talarodone A	C_20_H_23_NO_8_	405	[18]

**Table 2:** Fungal species/strains reported as producers of funicone-like compounds.

Species	Source/Lifestyle/Substrate	Location	Compounds	Ref.
*Dothideomycetes*, *Pleosporales*, *Didymellaceae*				
*Phoma* sp. nov. LG0217	Endophytic in *Parkinsonia microphylla*	Tucson (Arizona, USA)	**15, 18**	[36]
*Dothideomycetes*, *Botryosphaeriales*, *Phyllostictaceae*				
*Guignardia* sp. No. 4382	Endophytic in *Kandelia candel*	Hong Kong (China)	**17**	[49]
*Dothideomycetes*, *Mycosphaerellales*, *Mycosphaerellaceae*				
*Pseudocercospora (=Mycosphaerella) fijiensis*	Banana plant	Honduras	**15**	[30]
*Dothideomycetes*, *Capnodiales*, *Dissoconaceae*				
*Ramichloridium apiculatum* NHL2956	Air in bakery	Nagoya (Japan)	**11**	[27]
*Dothideomycetes*, *Cladosporiales*, *Cladosporiaceae*				
*Cladosporium* sp. JS1-2	endophytic in *Ceriops tagal*	Hainan (China)	**15**	[35]
*Eurotiomycetes*, *Eurotiales*, *Aspergillaceae*				
*Aspergillus neoglaber* (identified as *Neosartorya glabra*) CGMCC 32286	Unknown	China	**24**	[40]
*Aspergillus ruber* (identified as *Eurotium rubrum*) SH-823	Soft coral (*Sarcophyton* sp.)	Xuwen (China)	**15, 23**	[42]
*Penicillium citreonigrum* PAI 1/1 C	Sponge (*Pseudoceratina purpurea*)	Bali (Indonesia)	**3, 15, 18**	[12]
*Penicillium glabrum* SF-7123	Sediment	Ross Sea (Antarctica)	**3**	[13]
*Penicillium simplicissimum* IFM53375	Unknown	Japan	**1, 15, 16, 18, 20, 25**	[6]
*Penicillium* sp.	Endophytic in *Riccardia multifida*	Maoer Mountain (China)	**8, 17, 1**	[8]
*Penicillium* sp.	Unknown	Japan	**3**	[10]
*Penicillium* sp.	Unknown	Japan	**9**	[16]
*Penicillium* sp.	Ash	Mount Pinotubo (Philippines)	**3**	[11]
*Eurotiomycetes*, *Eurotiales*, *Trichocomaceae*				
*Talaromyces flavus*			**15**	[29]
*Talaromyces flavus* CCM-F748		Slovakia	**15**	[47]
*Talaromyces flavus* FKI-0076	Soil	Hiroo (Japan)	**2, 3, 15**	[9]
*Talaromyces flavus* IFM52668	Unknown	Japan	**1, 4, 15**	[4]
*Talaromyces pinophilus* F36CF	Endophytic in *Arbutus unedo*	Favignana Isle (Italy)	**10**	[58]
*Talaromyces pinophilus* H608	Mangrove sediment	Xiamen (China)	**1, 3, 10, 15, 21, 23**	[7]
*Talaromyces* sp. IPV2 (identified as *Penicillium funiculosum*)	Apple root	Sondrio Province (Italy)	**1**	[2,59]
*Talaromyces pinophilus* LT4, LT6	Soil from rhizosphere of *Nicotiana tabacum*	Lecce Province (Italy)	**7, 10**	[15,19]
*Talaromyces pinophilus* SCAU037	Soil from rhizosphere of *Rhizophora stylosa*	Techeng Isle (China)	**10, 12, 13, 15, 17, 18, 19, 21, 22, 23, 25, 26, 28, 29, 32, 33**	[28]
*Talaromyces pinophilus* ST2	Soil from rhizosphere of *Nicotiana tabacum*	Scafati (Italy)	**10**	[25]
*Talaromyces purpureogenus* MHZ 111	Soil	Mohe (China)	**15, 18**	[46]
*Talaromyces ruber* (identified as *Penicillium rubrum*)	Water	Berkeley Pit lake (USA)	**15, 18, 25**	[45]
*Talaromyces* sp. ZHS32	Marine sediment	Zhejiang (China)	**15**	[39]
*Talaromyces* sp. AF1-2 (unidentified in original report)	Salt pan	Australia	**10**	[26]
*Talaromyces* sp. HM6-1-1	Seawater	Dongshan Isle (China)	**15, 18**	[38]
*Talaromyces* sp. HN29-3B1 (identified as *Penicillium* sp.)	Endophytic in *Cerbera manghas*	Hainan (China)	**15, 17, 19, 21 23, 25, 26, 27**	[21]
*Talaromyces* sp. HSZ-43 (identified as *Penicillium* sp.)	Endophytic in *Trypterigium wilfordii*	Shanxi (China)	**1, 3, 10, 14**	[5]
*Talaromyces* sp. IFB-E022 (identified as *Penicillium* sp.)	Endophytic in *Quercus variabilis*	Zijin Mountain (China)	**30, 31, 32**	[51]
*Talaromyces* sp. XWS02F62 (identified as *Penicillium* sp.)	Sponge (*Callyspongia* sp.)	Xuwen County (China)	**15, 18**	[41]
*Talaromyces thailandiasis* KPFC 3399	Soil	Thailand	**15, 20, 25**	[33]
*Talaromyces verruculosus* CMI294548	Unknown	Pakistan	**15**	[31]

**Table 3:** Microbial species/strains reported as producers of funicone-like compounds in co-cultures.

Species 1	Species 2	Source/Substrate	Location	Compounds	Ref.
*Alternaria alternata* YX-25	*Streptomyces exfoliatus* YX-32	mangrove mud	Yunxiao (China)	**15**	[37]
*Penicillium* sp. WC-29-5	*Streptomyces fradiae* 007	rhizosphere of *Aegiceras corniculatum*/sediment	Hainan (China) Jiaozhou Bay (China)	**3, 5, 6, 15**	[14]
*Talaromyces pinophilus* 17F4103	*Paraphaeosphaeria* sp. 17F4110	soil	Miyazaki (Japan)	**10, 14, 32, 33, 34**	[18]
*Talaromyces siamensis* FKA-61	*Phomopsis* sp. FKA-62	soil	Japan	**15**	[43]

**Table 4:** Main bioactivities of funicone-like compounds.

Name (Code)	Bioactivity	Concentration	Bioassay	Ref.
Actofunicone (**2**)	Reinforcement of miconazole	3.7 µM	*Candida albicans* (IC_50_)	[9]
6-Demethylpenisimplicissin (**26**)	Enzyme inhibitory	9.5 µM	α-glucosidase (IC_50_)	[21]
Deoxyfunicone (**3**)	Anticholesterol	10 µM	Efflux from RAW264.7	[7]
	Antiviral	4.6 µM	HCV (IC_50_ on Huh-7.5.1)	[61]
	Cytotoxic	22.6 µM	KB (IC_50_)	[5]
	Enzyme inhibitory	24.3 µM	Protein tyrosine phosphate 1B (IC_50_)	[13]
		1.1–4.4 µM	HIV-1-integrase (IC_50_)	[11]
	Lipid inhibitory	10 µM	Accumulation in HepG2	[7]
			Downregulation of FAS, ACC, HMGR	
			Decrease in oxLDL in RAW264.7	
	NO inhibitory	10.6 µM 40.1 µM	LPS-stimulated BV2 (IC_50_) LPS-stimulated RAW264.7 (IC_50_)	[13]
	PGE_2_ inhibitory	32.3 µM	LPS-stimulated BV2 (IC_50_)	[13]
	Reinforcement of miconazole	1.6 µM	*C. albicans* (IC_50_)	[9]
2″-epiHydroxydihydrovermistatin (**19**)	Enzyme inhibitory	8 µM	α-glucosidase (IC_50_)	[21]
9,14-Epoxy-11-deoxyfunicone (**4**)	Antifungal	0.53 µmol/disc	*Aspergillus niger*	[4]
9*R*,14*S*-Epoxy-11-deoxyfunicone (**5**)	Cytotoxic	3.97 µM	H1975 (IC_50_)	[14]
9*S*,14*R*-Epoxy-11-deoxyfunicone (**6**)	Cytotoxic	3.73 µM 5.73 µM	HL-60 (IC_50_) H1975 (IC_50_)	[14]
Funicone (**1**)	Anticholesterol	10 µM	Efflux from RAW264.7	[7]
	Antifungal	0.27 µmol/disc	*Aspergillus fumigatus*	[4]
	Cytotoxic	13.2 µM	KB (IC_50_)	[5]
	Lipid inhibitory	10 µM	Accumulation in HepG2	[7]
			Downregulation of FAS, ACC, HMGR	
Isofunicone (**9**)	Pollen growth inhibitory	8.02 mM	*Camellia sinensis* (84%)	[16]
Hydroxyvermistatin (**21**)	Anticholesterol	10 µM	Efflux from RAW264.7	[7]
			Upregulation of PPARγ, LXRα, ABCG1	
			Decrease scavenger receptors CD36, SR-1	
	Enzyme inhibitory	20.3 µM	α-glucosidase (IC_50_)	[21]
	Lipid inhibitory	10 µM	Accumulation in HepG2	[7]
			Decrease in FAS, ACC, HMGR	
			Decrease in oxLDL in RAW264.7	
Methoxyvermistatin (**23**)	Anticholesterol	10 µM	Decrease scavenger receptors CD36, SR-1	[7]
	Cytotoxic	0.056 mM 0.042 mM	KB (IC_50_) KBv200 (IC_50_)	[34]
	Enzymatic inhibitory	236 µM	α-glucosidase (IC_50_)	[42]
	Lipid inhibitory	10 µM	Decrease in oxLDL in RAW264.7	[7]
3-*O*-Methylfunicone (**10**)	Anticholesterol	10 µM	Efflux from RAW264.7	[7]
	Antifungal	0.27 mM	*Rhizoctonia solani*, *Fusarium solani*, *Cylindrocladium scoparium*, *Alternaria alternata* (IC_100_)	[19]
	Antiviral	5 µM	decreased mortality of MDBK infected by BoHV-1	[62]
		6.2 µM	HCV (IC_50_ on Huh-7.5.1)	[61]
	Cytotoxic/ antiproliferative/ proapoptotic	35.3 µM	KB (IC_50_)	[5]
		10 µM	MDBK (IC_50_)	[62]
		63.8 µM 63.3 µM	HCT116 (LD_50_) HeLa (LD_50_)	[26]
		0.16 mM	HEp-2; inhibition colony formation, decrease neutral red uptake, inhibition O_2_ consumption (IC_50_)	[24]
		0.07 mM	HeLa (44%); promotion *p21*; downregulation cyclin D1/Cdk4 complex	[63]
		0.21 mM	MCF-7; downregulates αvβ5 integrin, MMP-9 inhibitor, impairs microtubule assemblage, inhibitor of *survivin*, *hTERT* and *Nanog-1* expression, reduces mammospheres	[64,65]
		0.21 mM	A375M (IC_85_, 48 h)	[66]
		0.14 mM	NCI-H2452; decreases αvβ5 integrin, MMP-2, VEGF, ERK1/2; synergism with cisplatin	[67,68]
	Enzyme Inhibitory	12.5 µM 50.1 µM 34.3 µM	DNA polymerase κ DNA polymerase η DNA polymerase ι	[26]
		5 mM	DNA polymerase κ and η	[52]
	Insecticidal	0.14 mM	*Acyrthosiphon pisum* (26.2%)	[23]
	Lipid inhibitory	10 µM	Accumulation in HepG2	[7]
			Decrease in FAS, ACC, HMGR	
			Decrease in oxLDL in RAW264.7	
Penicidone A (**30**)	Cytotoxic	60.1 µM 54 µM 46.5 µM 41.5 µM	SW116 (IC_50_) K562 (IC_50_) KB (IC_50_) HeLa (IC_50_)	[51]
Penicidone B (**31**)	Cytotoxic	54.2 µM 21.1 µM 29.6 µM 35.1 µM	SW116 (IC_50_) K562 (IC_50_) KB (IC_50_) HeLa (IC_50_)	[51]
Penicidone C (**32**)	Cytotoxic	80.8 µM 54.3 µM 44.3 µM 54.7 µM	SW116 (IC_50_) K562 (IC_50_) KB (IC_50_) HeLa (IC_50_)	[51]
	Enzyme inhibitory	51.9 µM	α-glucosidase (IC_50_)	[28]
Penifupyrone (**14**)	Cytotoxic	4.7 µM	KB (IC_50_)	[5]
Penisimplicissin (**25**)	Cytotoxic	−6.70 −5.83	CCRF-CEM (log_10_ GI_50_) HL-60 (log_10_ GI_50_)	[45]
	Enzyme inhibitory	0.66 mM 0.33 mM	IL-1β (IC_100_) caspase 1 (IC_100_)	[44]
Rapicone (**11**)	Enzyme inhibitory	5 mM	DNA polymerase κ	[52]
Vermistatin (**15**)	Antibacterial	0.076 mM	*Staphylococcus aureus*, *Bacillus cereus* (MIC)	[35]
	Anticholesterol	10 µM	Efflux from RAW264.7	[7]
			Decrease scavenger receptors CD36, SR-1	
	Cytotoxic	0.28 mM	KB (IC_50_)	[34]
		33.9 µM	B16 (IC_50_)	[39]
	Enzyme inhibitory	29.2 µM	α-glucosidase (IC_50_)	[21]
		107.1 µM	α-glucosidase (IC_50_)	[42]
	Insecticidal	0.46 mM	*Helicoverpa armigera* (IC_50_)	[35]
	Lipid inhibitory	10 µM	accumulation in HepG2	[7]
			Decrease in FAS, ACC, HMGR	
			Decrease in oxLDL in RAW264.7	
	NO inhibitory	52.7 µM	LPS-stimulated BV2 (IC_50_)	[46]
	Phytotoxic	3.1–6.1 mM	Banana leaves	[30]
	Reinforcement of miconazole	2.1 µM	*C. albicans* (IC_50_)	[9]

## Data Availability

Not applicable.
